# HPV Type Distribution and Cervical Cytology among HIV-Positive Tanzanian and South African Women

**DOI:** 10.5402/2012/514146

**Published:** 2012-06-28

**Authors:** Joke A. M. Dols, Gregor Reid, Joelle M. Brown, Hugo Tempelman, Tj. Romke Bontekoe, Wim G. V. Quint, Mathilde E. Boon

**Affiliations:** ^1^Erasmus MC, University Medical Center, P.O. Box 2040, 3000 CA Rotterdam, The Netherlands; ^2^Leiden Cytology and Pathology Laboratory, P.O. Box 16084, 2301 GB Leiden, The Netherlands; ^3^Lawson Health Research Institute, Canadian Research and Development Centre for Probiotics and Departments of Microbiology, Immunology and Surgery, University of Western Ontario, London, Canada N6A 4V2; ^4^Department of Epidemiology, University of California Los Angeles, Los Angeles, CA 90095, USA; ^5^Ndlovu Care Group, Elandsdoorn, P.O. Box 15008, Groblersdal 0470, South Africa; ^6^Bontekoe Research, Rooseveltstraat 4-d, 2321 BM Leiden, The Netherlands; ^7^DDL Diagnostic Laboratory, Visseringlaan 25, 2288 ER Rijswijk, The Netherlands

## Abstract

*Background*. There are limited data on high-risk human papillomavirus (hr-HPV) genotypes among HIV-positive women in Africa, and little is known about their relationship with cervical cytology in these populations. *Methods*. We conducted a cross-sectional study among 194 HIV-positive women (143 from Tanzania, and 51 from South Africa) to evaluate HPV genotypes among HIV-positive women with normal and abnormal cytology. Cervical samples were genotyped for HPV types, and slides were evaluated for atypical squamous cell changes according to the Bethesda classification system. *Results*. Prevalence of high grade squamous intraepithelial dysplasia (HSIL) was 9%. Overall, more than half (56%) of women were infected with an hr-HPV type; 94% of women with HSIL (*n* = 16), 90% of women with LSIL (*n* = 35), and 42% of women within normal limits (WNL) (*n* = 58) tested positive for hr-HPV. Overall, the most prevalent hr-HPV subtypes were HPV16 (26%) and HPV52 (30%). Regional differences in the prevalence of HPV18 and HPV35 were found. *Conclusion*. Regional differences in HPV genotypes among African women warrant the need to consider different monitoring programmes for cervical preneoplasia. HPV-based screening tests for cervical preneoplasia would be highly inefficient unless coupled with cytology screening of the HPV-positive sample, especially in HIV-positive women.

## 1. Introduction

Cervical carcinoma is the second most prevalent cancer in the world, and the most common female cancer in sub-Saharan Africa. Infection with high-risk human papillomavirus (hr-HPV) is the main risk factor for the development of cervical carcinoma. HIV-positive women are reported to be almost twice as likely to be concurrently infected with HPV than HIV-negative women. Recently introduced HPV vaccines against genotypes 16 and 18 are designed to prevent about 70% of cervical cancers. The basis for selecting these genotypes was their prevalence in Europe and North America [1]. The relevance of these genotypes to women in other parts of the world has been questioned, with a study of HPV genotypes in women in 38 countries including some in Central America, Africa, Asia, and Oceania [2]. In this study, hr-HPV types 16 and 18 were detected in 71% of cases of invasive cervical cancer. Of the cases of invasive cervical cancer from Africa, hr-HPV 16 and 18 were detected, respectively, in 48% and 23% of cases that were positive for HPV DNA. 

There are limited data on hr-HPV genotypes among HIV-positive women in Africa, and little is known about their relationship with cervical cytology in these populations. We carried out a study to determine the prevalence of HPV genotypes among HIV-positive women in Africa with normal and abnormal cervical cytology scores. 

## 2. Methods

### 2.1. Subjects

Cervical samples were collected from women attending the HIV treatment clinic of Sekou-Toure regional hospital and surrounding hospitals in Mwanza, Tanzania between February and March 2010, as part of a randomized controlled trial. Eligible subjects were nonpregnant, HIV-positive females, over 18 years of age who had used antiretroviral therapy (ART) for at least 6 months. Subjects were excluded if pregnant, breastfeeding, intolerant to lactose or fermented milk. In total 168 Tanzanian women were included in the study, of whom 143 women had cervical swabs adequate for HPV testing at baseline. The Medical Research Coordinating Committee of the National Institute for Medical Research, Tanzania, approved the study design and protocol. Participants were informed of the purpose of the study and gave their signed or thumb-printed informed consent before participation (ClinicalTrials.gov Identifier: NCT01258556) [3]. 

Cervical samples collected in 2008 from HIV-positive women attending the Ndlovu Medical Centre in Elandsdoorn in Moutse District, South Africa, were also included in this analysis. In total, 51 women testing positive for HIV were included. Information was provided to the study participants and oral consent was obtained by the doctors for a cervical sample to be examined. Pregnant women and those aged ≤16 years were excluded. None of the HIV-positive women were receiving ART at the time of sample collection. 

Cervical samples from all participants were collected by a clinician using a standard sampling brush, and placed into a vial with coagulant fixative Boonfix, which consists of ethyl alcohol, low molecular weight PEG, and acetic acid. This substance has no detrimental effects on DNA preservation or DNA isolation [4]. The fixed samples, coded by a method that did not disclose any subject names, were sent to the Leiden Cytology and Pathology Laboratory (LCPL) in The Netherlands for analysis [5, 6].

### 2.2. Cytology Classification

A ThinPrep Cytology slide was made of the Boonfixed samples, and stained with the Papanicolaou method. The Cytology ThinPrep slides were classified according to the Bethesda classification system as WNL (within normal limits), ASCUS or LSIL (atypical squamous cells of undetermined significance and low-grade squamous intraepithelial lesions), or HSIL (high-grade squamous intraepithelial lesions). Of the cases with an abnormal cytology score (ASCUS, LSIL, and HSIL), the residue of the cervical sample was used to prepare histologic paraffin slides [7, 8]. Biopsy confirmation could not be performed.

### 2.3. HPV Genotyping

The purification of DNA from the Boonfix solution was performed in the BioRobot M48, Qiagen (Germany), which uses the MagAttract DNA Mini M48 Kit. This magnetic-particle technology provides high-quality DNA. Samples were analyzed using a sensitive SPF 10 PCR-reverse hybridization line probe assay (Innogenetics, Belgium). This system is based on PCR of part of the L1 region of the HPV genome, which is amplified using SPF10 primers [9]. Detection after PCR is based on the principle of reverse hybridization. Amplification products are subsequently hybridized using specific oligonucleotide probes in a single typing strip. This strip included 14 hr-HPV types (16, 18, 31, 33, 35, 39, 45, 51, 52, 56, 58, 59, 66, and 68) [1]. Low risk (lr) and undetermined HPV types are differentiated into 13 types (6, 11, 26, 40, 43, 44, 53, 54, 69, 70, 73, 74, and 82).

### 2.4. Data Analysis

Type-specific HPV prevalence was compared between women with HSIL, LSIL, and WNL. The chi-square test was used to test for significant associations between country and HPV type. Analyses were conducted using SPSS and Mathematica. 

## 3. Results

### 3.1. Cervical Cytology

**Figure 1:**
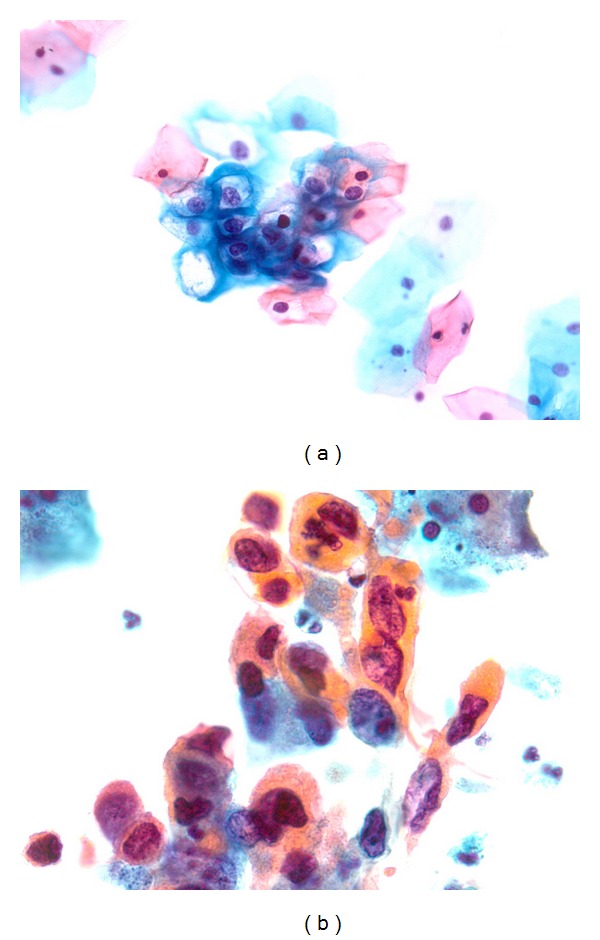
(a) LSIL, with polyploid (enlarged) nuclei and koilocytosis. Tested positive for hr-HPV 52 and hr-HPV 56. (b) HSIL, dyskeratotic cells. Tested positive for lr-HPV 44, hr-HPV 52, and undetermined HPV types.

One hundred three (72%) Tanzanian women and 35 (69%) South African women had a normal cytology score (WNL) (Table 1). Nine (6%) Tanzanian women and 8 (16%) South African women had HSIL. Examples of cases with an abnormal cytology score are presented in Figure 1. 

### 3.2. HPV Genotypes

**Figure 2:**
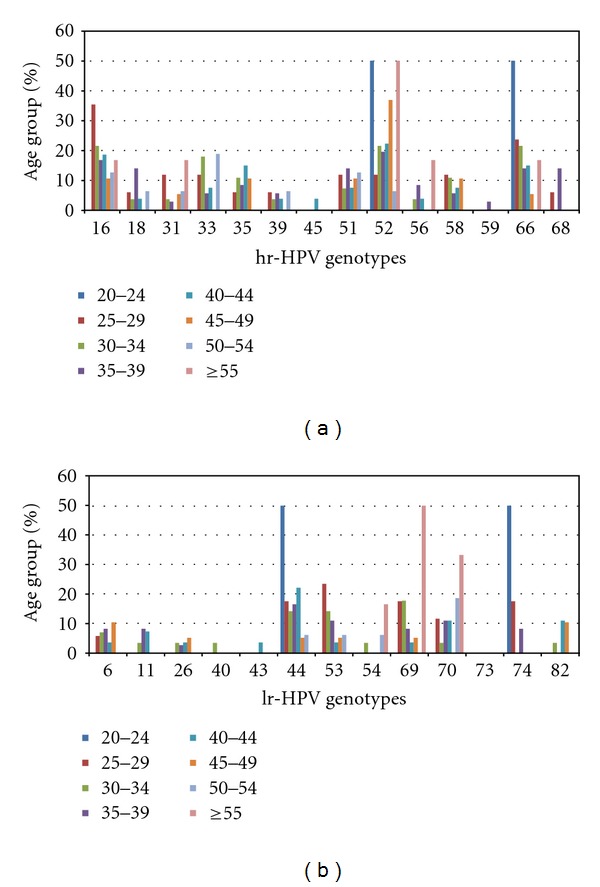
(a) Age distribution versus hr-HPV genotypes. (b) Age distribution versus lr-HPV genotypes.

 Overall, 109 (56%) women tested positive for hr-HPV (Table 2); 80 (56%) Tanzanian women and 29 (57%) South African women. Seventy-six (53%) Tanzanian women and 30 (59%) South African women tested positive for a lr-HPV or undetermined HPV type. Among women testing positive for HPV, the mean age was 35.4 years (range: 20–52) in South Africa and 40.5 years (range: 25–73) in Tanzania. The age distribution per HPV genotype is shown in Figure 2. 

**Figure 3:**
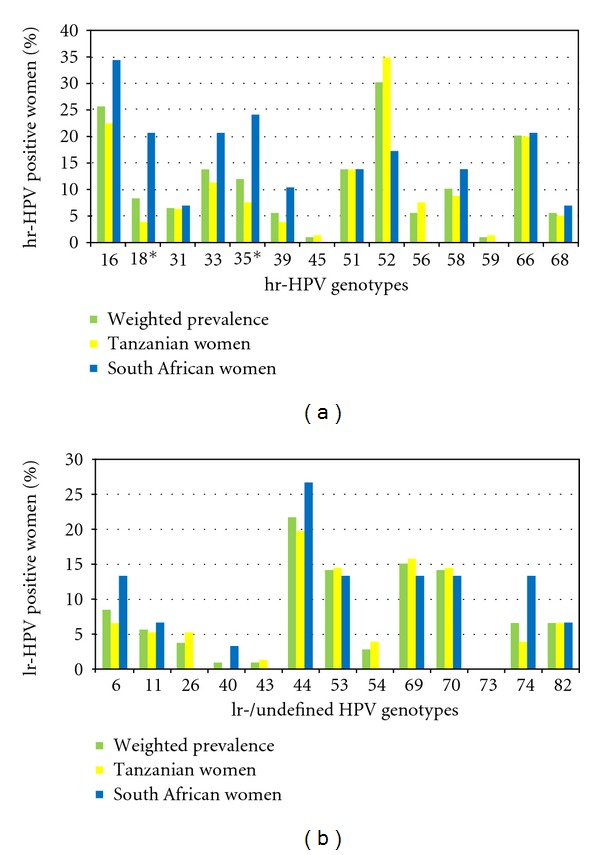
(a) Percentage weighted prevalence with country specific hr-HPV prevalence is shown. Significant differences between Tanzania and South Africa are marked with an asterisk if *P* < 0.05. (b) Percentage weighted prevalence with country specific lr-HPV prevalence is shown.

 Overall, the most prevalent hr-HPV subtypes were hr-HPV 16 (26%) and hr-HPV 52 (30%), and the prevalence of hr-HPV genotypes varied by country. Among Tanzanian women testing positive for any hr-HPV, the most common genotype was hr-HPV 52 (35%), followed by hr-HPV 16 (23%) and hr-HPV 66 (20%). In contrast, among South African women testing positive for any hr-HPV, the most common genotype was hr-HPV 16 (34%), followed by hr-HPV 35 (24%), hr-HPV 18 (21%), hr-HPV 33 (21%), and hr-HPV 66 (21%). Genotypes 18 and 35 were significantly more common in South Africa than Tanzania; the prevalence of genotype 18 was 21% in South Africa compared to 4% in Tanzania (*P*-value = 0.01), and the prevalence of hr-HPV 35 was 24% in South Africa and 8% in Tanzania (*P*-value = 0.03) (Figure 3(a)). The most prevalent lr-HPV subtype overall was hr-HPV 44 (22%) (Figure 3(b)).

### 3.3. Association between Cervical Cytology and HPV Genotype

All women with abnormal cytology (ASCUS, LSIL, or HSIL) had detectable HPV (Table 2). Ninety-four percent (16/17) of women with HSIL, 90% (35/39) of women with LSIL, and 42% (58/138) of women within normal limits (WNL) tested positive for hr-HPV. Women with HSIL were more likely to test positive for hr-HPV 16 compared to women with normal cytology (WNL) (29% versus 6%, *P* < 0.01). Hr-HPV 18 was found in only 11.8% of women with HSIL (Table 3) and the prevalence varied by country; the prevalence of hr-HPV 18 was 4% in Tanzania compared to 21% in South Africa (*P*-value = 0.01).

## 4. Discussion

 This study found that hr-HPV genotypes not included in the currently licensed HPV vaccines are highly prevalent in HIV-positive women from Tanzania and South Africa. As expected, among women with hr-HPV the prevalence of hr-HPV 16 was high (26%). 

 Despite the high rate of abnormal cytology among women in Tanzania, hr-HPV 18 was uncommon in women with abnormal cytology (4%). In contrast, hr-HPV 18 was significantly more common among South African women with abnormal cytology (21%). We also found significant regional differences in the prevalence of hr-HPV 35; the prevalence of hr-HPV 35 was 8% among Tanzanian women compared to 24% for South African women. In rural Mozambique, Castellsagué et al. also found a high prevalence of hr-HPV 35 (17% among HPV-positive women, and 30% among women with cervical neoplasia [10]), compared to an hr-HPV 35 prevalence of 5% found by Sanjose in other African women with cervical neoplasia (Algeria, Mozambique, Nigeria, and Uganda) [2].

The hr-HPV 52 prevalence found in this study (35% of Tanzanian women and 17% of South African women with any HPV) is vastly different from that in women in The Netherlands (5.5%) [11]. A study in Pretoria, South Africa reported an hr-HPV 52 prevalence of 6% among women with cervical preneoplasia [12]. In the general female population in Macao, and northern Taiwan, hr-HPV 52 was the most common genotype found, while in East Asia it was among the top five detected [13–15].

The prevalence of hr-HPV 45 was low in our cohort (Figure 3(a)), as also found by others in women with preneoplastic lesions [2]. However, hr-HPV 45 is the third most common HPV type in invasive cervical cancer worldwide. The early presentation of cases of invasive cervical cancer that are positive for hr-HPV 45 might be related to a short time for progression to invasive cancer, with or without transition through the preneoplastic stages, possible correlated to a high early integration rate [2]. 

The strengths of our study include using a standardized HPV type-specific PCR protocol and cytology diagnosis across the two sites. There were differences between the women studied that may have contributed to the different prevalence estimates of hr-HPV types. Tanzanian women were all using ART. In contrast, the women in South Africa were recently diagnosed with HIV and not yet eligible for ART. In HIV positive women there is less HPV clearance due to a lack of an appropriate immune response. The impact of ART in HPV infection and cervical cancer still needs to be defined [16]. A limitation of this study is that no additional information from the South African women is known about their sexual behavior, and therefore we were unable to explore associations between behaviors and HPV genotype. Roteli-Martins et al. found that young age at first sexual intercourse and increasing number of sexual partners is the main determinant for hr-HPV 16/18 infections [17]. 

For an HPV vaccine to effectively reduce incidence of cervical cancer, it will need to protect against the major hr-HPV infection types progressing to cervical cancer. Because infection types may vary across settings and ethnicities, the impact a vaccine has in preventing cancer in particular settings needs to be considered. In this study, hr-HPV16 was found in 29% of women diagnosed with HSIL; however, hr-HPV 18 was only found in 11%.

Certainly the high prevalence of hr-HPV found here and reported by others, [18] emphasizes the importance of screening for cervical cancer especially in developing countries with high rates of HIV. Because more than one-third (42%) of women with normal cytology in this study tested positive for an hr-HPV type, as also found by Bruni and others [19], HPV-based screening tests in this population would be highly inefficient unless coupled with cytology screening of the HPV positive sample. 

In summary, the present study showed some differences in the prevalence of HPV genotypes among HIV-positive women in two African countries, and an overall high HPV prevalence. The differences found in hr-HPV genotypes warrant the need to consider different monitoring programmes for cervical preneoplasia, especially in HIV-positive women. 

## Figures and Tables

**Table 1:** Cervical cytology results, overall and by country.

	Tanzanian women (*n* = 143)	South African women (*n* = 51)	Total (*n* = 194)
Negative Cytology (WNL)	103 (72.0%)	35 (68.6%)	138 (71.1%)
ASCUS-LSIL	31 (21.7%)	8 (15.7%)	39 (20.1%)
HSIL	9 (6.3%)	8 (15.7%)	17 (8.8%)

**Table 2:** Cytology score versus HPV testing.

	Negative cytology (WNL)	ASCUS-LSIL	HSIL	Total
Hr-HPV positive	58 (42.0%)	35 (89.7%)	16 (94.1%)	109 (56.2%)
Lr-HPV positive	68 (49.3%)	27 (69.2%)	11 (64.6%)	106 (54.6%)
HPV negative	43 (31.2%)	0	0	43 (22.2%)

Total	138 (71.1%)	39 (20.1%)	17 (8.8%)	194

**Table 3:** Cytology score versus hr-HPV.

	Negative cytology (WNL) (Total: 138)	ASCUS-LSIL (Total: 39)	HSIL (Total: 17)
Hr-HPV 16	8 (5.8%)	15 (38.5%)	5 (29.4%)
Hr-HPV 18	6 (4.3%)	1 (2.6%)	2 (11.8%)
Hr-HPV 31	5 (3.6%)	2 (2.9%)	0
Hr-HPV 33	11 (8.0%)	3 (7.7%)	1 (5.9%)
Hr-HPV 35	6 (4.3%)	3 (7.7%)	4 (23.5%)
Hr-HPV 39	3 (2.2%)	1 (2.6%)	2 (11.8%)
Hr-HPV 45	1 (0.7%)	0	0
Hr-HPV 51	10 (7.2%)	3 (7.7%)	2 (11.8%)
Hr-HPV 52	17 (12.3%)	9 (23.1%)	7 (41.2%)
Hr-HPV 56	3 (2.2%)	3 (7.7%)	0
Hr-HPV 58	3 (2.2%)	8 (20.5%)	0
Hr-HPV 59	0	1 (2.6%)	0
Hr-HPV 66	7 (5.1%)	13 (33.3%)	2 (11.8%)
Hr-HPV 68	2 (1.4%)	2 (2.9%)	2 (11.8%)
